# Conduction properties of the preferential pathway in a patient with idiopathic outflow ventricular arrhythmia

**DOI:** 10.1016/j.ipej.2021.12.002

**Published:** 2021-12-20

**Authors:** Yousaku Okubo, Sho Okamura, Takehito Tokuyama, Yukiko Nakano

**Affiliations:** Department of Cardiovascular Medicine, Hiroshima University Graduate School of Biomedical and Health Sciences, Hiroshima, Japan

**Keywords:** Ventricular arrhythmias, Premature ventricular contraction, Preferential pathway, High-density 3D mapping, Catheter ablation

## Abstract

Ventricular arrhythmias (VA) originating from the outflow tract often conduct via a preferential pathway into a distant breakout site in the ventricular myocardium. Preferential pathway potentials, characterized as presystolic potentials preceding the QRS onset during VA and late potentials during sinus rhythm, are known targets of successful cardiac ablation. However, the mechanism of conduction and properties of the preferential pathway has not yet been fully elucidated. In the present case, we evaluated the conduction properties of the preferential pathway using 3D electrical mapping in a patient with VA originating from the left ventricular outflow tract. Similar to the embryonic cardiomyocyte, slow conduction velocity, decremental property, and automaticity were found in the preferential pathway. Thus, the preferential pathway may be considered a remnant of the developing conduction system as so-called “dead end tract,” rather than the typical structures such as the LV myocardium or Purkinje tissue.

## 1 Introduction

Ventricular arrhythmias (VA) originating from the outflow tract often conduct via a preferential pathway into a distant breakout site within the ventricular myocardium. Several previous studies reported that ablation of preferential pathway potentials, characterized as presystolic potentials preceding the QRS onset during VA and late potentials during sinus rhythm, was effective therapy to treat outflow VA [1,2]. However, the mechanism(s) of conduction and properties of the preferential pathway have not yet been fully elucidated. In the present case, we evaluated the conduction properties of the preferential pathway in a patient with idiopathic premature ventricular contraction (PVC) originating from the left ventricular outflow, which provides new insight into the mechanisms of the preferential pathway and PVCs.

## 2 Case report

**Fig. 1:**
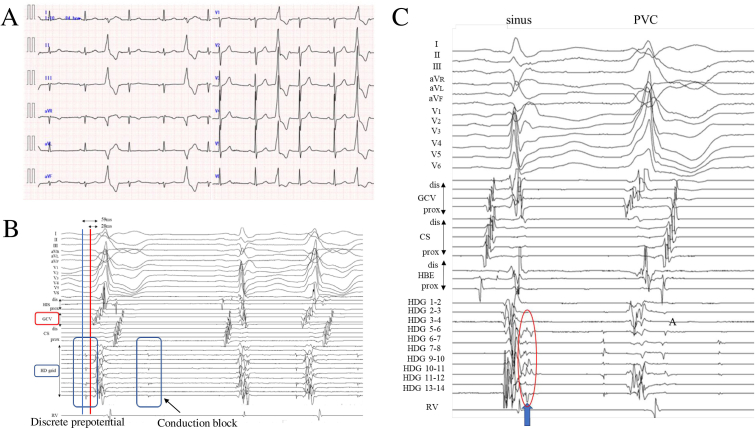
**A**: A 12-lead electrocardiogram showed regular sinus rhythm and frequent PVCs. The PVC showed right bundle branch block (RBBB) morphology, inferior axis, and the maximum deflection index (MDI) in the inferior lead of 0.57. Fig. 1B: The local ventricular activation time recorded at the great cardiac vein (GCV) preceded the onset of the QRS complex by 28 ms, and pace mapping at this site was a good match for the QRS complex of the clinical PVC. Discrete potentials preceding the QRS onset during PVC and non-conducted potential were recorded at the HD mapping catheter located at the LVOT. Fig. 1C: At the earliest site of the pre-potential during the PVC, late potentials were recorded during sinus rhythm (indicated by blue arrow and red circle).

A 76-year-old man with normal LV function presented with palpitations and high frequency (26%) monomorphic premature ventricular contractions (PVCs, 24-h Holter recording). His past medical history included paroxysmal atrial fibrillation that was treated by radiofrequency catheter ablation. A 12-lead electrocardiogram showed sinus rhythm and frequent PVCs (Fig. 1A). Clinical PVCs showed right bundle branch block (RBBB) morphology, inferior axis, and the maximum deflection index (MDI) in the inferior lead of 0.57.

In the electrophysiological examination, we placed a 2F octopolar electrode catheter (EPstar Fix AIV, Japan Lifeline, Tokyo, Japan) in the anterior interventricular vein (AIV) and the great cardiac vein (GCV) by using a 0.14-inch guidewire, a 5-Fr decapolar electrode catheter (Supreme™, Abbott, St. Paul, MN) at the His bundle site and an HD mapping catheter (Advisor™ HD Grid Mapping Catheter, Abbott, St. Paul, MN) in the RV outflow (RVOT). First, we performed the local activation time（LAT) mapping of PVCs at the RVOT and pulmonary artery by using the HD mapping catheter and EnSite™ Precision™ systems (Abbott, St. Paul, MN). However, since there was no location with earlier activation other than the GCV, we created a LAT map using the HD mapping catheter in the LV outflow tract (LVOT) and the aortic cusp. The local ventricular activation time recorded at the GCV preceded the onset of the QRS complex by 28 ms, and pace mapping at this site was highly consistent with the QRS complex of the clinical PVC. Discrete potentials preceding the QRS onset during PVC were recorded at the HD mapping catheter, which is located in the LVOT. It is often the case that similar artifact potentials are observed in the LVOT. In the present case, the discrete prepotentials were observed just before the QRS of the PVC, regardless of the timing of opening and closing of the aortic valve. Therefore, prepotential was not considered to be artifact of valve movement.

**Fig. 2:**
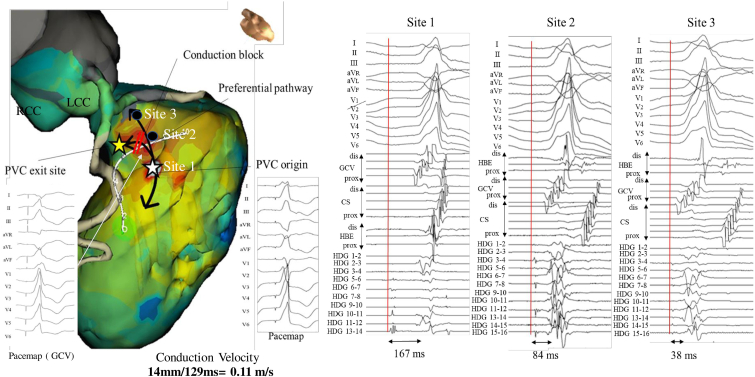
The discrete potentials preceding the QRS were recorded by the HD mapping catheter (Advisor™ HD Grid Mapping Catheter, Abbott, St. Paul, MN). These prepotentials were recorded to conduct longitudinally from the distal to the basal side of the LVOT. The earliest activation site (Site 1) is shown by a white star, and the VA exit site is shown by a yellow star. The impulse is conducted through the preferential pathway indicated by black allows. The conduction velocity of the preferential pathway was calculated by using a 3D mapping system and found to be extremely slow, 0.11 m/s. The pace map at site1, the PVC origin site, show extremely short stimulus-QRS interval in contrast to 167 ms EGM to QRS of native PVC. Although the pace map from the catheter located in the GCV indicated by the yellow star shows a perfect pace map (12/12) with the clinical PVCs, all the other pace maps from the endocardial site, including the earliest activation site (indicated by a white star) during the PVCs did not match.

Occasionally, the discrete potential was blocked and did not conduct (Fig. 1B). Furthermore, these prepotentials are conducted longitudinally from the apex to the basal side of the LVOT. The prepotential at the earliest activation site (EAS) in LVOT preceded the QRS onset by 167 ms during the PVCs. At this site, late potentials were recorded during sinus rhythm (Fig. 1C). Fig. 2 shows the schematic diagram of the PVCs. Although the pace map from the catheter located in the GCV indicated by the yellow star shows a perfect pace map (12/12) with the clinical PVCs, all the other pace maps from the endocardial site, including the earliest activation site (indicated by a white star) during the PVCs did not match. The pace map at site1, the PVC origin site, show extremely short stimulus-QRS interval in contrast to 167 ms EGM to QRS of native PVC. Since the pacing from the origin of the PVCs often does not selectively capture the preferential conduction, the pace map often does not match. This highlights potential shortcoming of pace-mapping.

The conduction velocity of the preferential pathway was calculated by dividing the distance from site1 to site3, 14mm, by the time taken for conduction using a 3D mapping system and found to be extremely slow, 0.11 m/s.

**Fig. 3:**
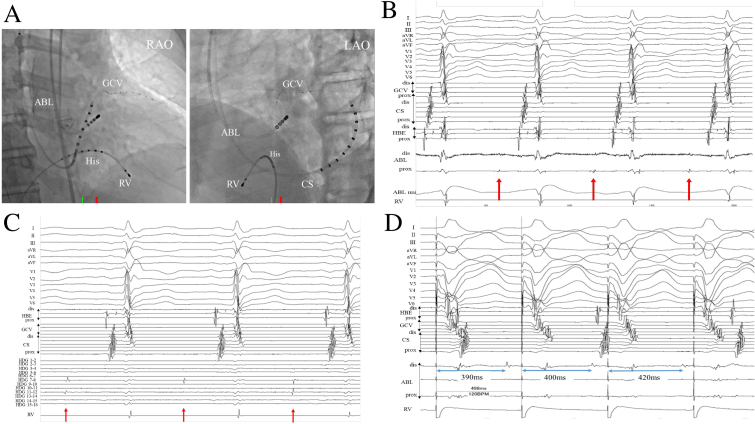
**A**: An 8F, 4 mm flexible irrigated-tip catheter (TactiCath™ Ablation Catheter, Abbott, St. Paul, MN) was positioned at the earliest activation site of prepotentials. The radiofrequency energy application between the VA origin and the VA exit immediately terminated the PVCs. Fig. 3B and C: The discrete potentials indicated by the red allow recordings by both the ablation and HD mapping catheter. Fig. 3D: The discrete potential was dependent on the RV pacing cycle (Pacing cycle length = 500 ms), which suggested that this potential was not completely isolated or bidirectionally blocked. Furthermore, the discrete potential exhibited Wenckebach-like behavior.

We positioned an 8-F, 4 mm flexible irrigated-tip catheter (TactiCath™ Ablation Catheter, Abbott, St. Paul, MN) at the earliest activation site (Fig. 3A), and then applied a radiofrequency (RF) energy application (40 W) to that site, eliminating the PVC in 2.3 s.

The intracardiac electrocardiogram collected after initial ablation is shown in Fig. 3B and C. The discrete potentials indicated by the red allow recorded by both the ablation catheter and the HD mapping catheter. Since the discrete potential was dependent on the RV pacing cycle, this potential was considered to be a late potential during sinus rhythm. Furthermore, the discrete potential exhibited Wenckebach-like behavior by incremental RV pacing (Fig. 3D). In the present case, we have shown that the preferential conduction pathway has an extremely slow conduction velocity, a decremental property, and automaticity.

## 3 Discussion

Several previous reports have demonstrated that discrete prepotentials during the VAs and late potentials during sinus rhythm could be successful ablation sites to treat VAs originating in the outflow tract [1,2]. Although the origin of the prepotentials is not fully elucidated, remnants of the developing conduction system at the LV outflow tract, a so-called “dead-end tract,” is speculated to consist of these potentials [3,4].

The two novel findings of this case report are 1) the preferential pathway runs longitudinally through the LV and fades out at the top of the LV summit below the attachment of the aortic valve leaflets. Immediately after applying RF energy to the EAS, a conduction block between the VA origin and VA exit site occurred, and the VA was terminated. 2) The conduction velocity of the preferential pathway was extremely slow and different from that of ventricular muscle and Purkinje fibers. Previous studies demonstrated that the conduction velocity of Purkinje fibers was 2 m/s - 4 m/s and that of ventricular muscle cell was 0.4 m/s - 1.0 m/s. Moreover, it is conductive and shares automatic properties [5]. These conduction characteristics of the preferential pathway were similar to those of embryonic cardiomyocytes rather than the usual structures such as the LV myocardium or Purkinje tissue.

To the best of our knowledge, this is the first case showing preferential conduction direction and velocity in a patient with VA using a 3D mapping system.

## Declaration of competing interest

None.

## References

[1] Yamada T., Litovsky S.H., Kay G.N. (2008). The left ventricular ostium:an anatomic concept relevant to idiopathic ventricular arrhythmias. Circ Arrhythm Electrophysiol.

[2] Yamada T., Murakami Y., Yoshida N. (2007). Preferential conduction across the ventricular outflow septum in ventricular arrhythmia originating from the aortic sinus cusp. J Am Coll Cardiol.

[3] Kurosawa H., Becker A.E. (1985). Dead-end tracts of the conduction axis. Int J Cardiol.

[4] Wessels A., Vermeulen J.L., Vira'gh S. (1991). Spatial distribution of “tissue-specific” antigens in the developing human heart and skeletal muscle. III.An immunohistochemical analysis of the distribution of the neural tissue antigen GlN2 in the embryonic heart; implications for the development of the atrioventricular conduction system. Anat Rec.

[5] Scher A.M., Spach M.S., Berne R.M. (1979). The cardiovascular system.

